# Ultrasound at labour triage in eastern Uganda: A mixed methods study of patient perceptions of care and providers’ implementation experience

**DOI:** 10.1371/journal.pone.0259770

**Published:** 2021-11-12

**Authors:** Nathan Isabirye, Rose Kisa, Nicole Santos, Sachita Shah, Jude Mulowooza, Dilys Walker, Peter Waiswa

**Affiliations:** 1 School of Public Health, Makerere University, Kampala, Uganda; 2 Institute for Global Health Sciences, University of California San Francisco, San Francisco, California, United States of America; 3 Department of Emergency Medicine, University of Washington, School of Medicine, Seattle, WA, United States of America; 4 Department of Obstetrics, Gynaecology & Reproductive Sciences, University of California San Francisco, San Francisco, California, United States of America; University of Mississippi Medical Center, UNITED STATES

## Abstract

In settings where antenatal ultrasound is not offered routinely, ultrasound use when a woman first presents to the maternity ward for labour (i.e., triage) may be beneficial. This study investigated patients’ perceptions of care and providers’ experience with ultrasound implementation during labour triage at a district referral hospital (DH) and three primary health centers (HC) in eastern Uganda. This was a mixed methods study comprising questionnaires administered to women and key informant interviews among midwives pre- and post-ultrasound introduction. Bivariate analyses were conducted using chi-square tests. Qualitative themes were categorized as (1) workflow integration; (2) impact on clinical processes; (3) patient response to ultrasound; and (4) implementation barriers. A total of 731 and 815 women completed questionnaires from the HCs and DH, respectively. At the HC-level, triage quality of care, satisfaction and recommendation ratings increased with implementation of ultrasound. In contrast, satisfaction and recommendation ratings did not differ upon ultrasound introduction at the DH, whereas perceived triage quality of care increased. Most participants noted a perceived improvement in midwives’ experience and knowledge upon introduction of ultrasound. Women who underwent a scan also reported diverse feelings, such as fear or worry about their delivery, fear of harm due to the ultrasound, or relief after knowing the baby’s condition. For the midwives’ perspective (n = 14), respondents noted that ultrasound led to more accurate diagnoses (e.g., fetal position, heart rate, multiple gestation) and improved decision-making. However, they noted health system barriers to ultrasound implementation, such as increased workload, not enough ultrasound-trained providers, and irregular electricity. While triage ultrasound in this context was seen as beneficial to mothers and useful in providers’ clinical assessments, further investigation around provider-patient communication, system-level challenges, and fears or misconceptions among women are needed.

## Introduction

Utilization of obstetric ultrasound in many low-and middle-income countries (LMICs) has increased due to improvements in machine durability, portability, and affordability [1–5]. Previous studies in LMICs indicate that trained midwives have the capacity to diagnose different obstetric conditions using ultrasound during pregnancy [6,7]. For example, a maternal ultrasound-training program was offered to 21 midwives in a rural health district in Zambia [8]. Quality assurance related to placental location and fetal heart interpretation showed greater than 90% agreement between learners and external reviewers. Furthermore, 17% of scans prompted a change in clinical decision-making, such as increased antenatal care visits, referral, admission for observation, or labour induction. In another study, 14 health centre midwives in rural southwest Uganda were trained over a 6-week period to conduct limited, screening obstetric ultrasound. They demonstrated that ultrasound corrected clinical exam findings 6.7% to 12% of the time, including identification of early pregnancy complications (e.g., ectopic pregnancy, incomplete/compete abortion), as well as malpresentation and multiple gestation [9]. Thus, midwife-administered ultrasound may be valuable in early and accurate diagnosis of certain conditions during pregnancy, and subsequent clinical decision-making.

Despite the utility of obstetric ultrasound, its implementation in some African settings has been characterized by challenges to both providers and patients. In Rwanda, midwives reported differing views on the use of ultrasound, unequal access in rural versus urban areas, and expressed a desire that they be trained in order to reduce reliance on radiologists and sonographers [10]. Another study among providers in Tanzania showed that although ultrasound was perceived as necessary for proper management of pregnancy complications; however, concerns related to limited availability of ultrasound, lack of trained staff, and difficulties counselling patients were expressed [11,12]. From the patient perspective, both positive and negative perceptions have been reported regarding obstetric ultrasound among women in Nigeria, Zambia, Botswana and Kenya [13]. Some women believed ultrasound provided motivation to attend antenatal care [8], and reassurance in terms of knowing the status of their pregnancy [14,15]. Others, however, viewed ultrasound unfavourably, expressing fear of the ultrasound scan, or harm to their foetus [13,14,16].

The World Health Organization (WHO) recommends early ultrasound before 24 weeks of gestation in all women to ensure a positive pregnancy experience [17]. Similarly, Uganda’s protocol for focused antenatal care recommends ultrasound between 20 to 24 weeks for women attending their second or third antenatal care visit. However, in many public rural facilities, antenatal ultrasound is often not routinely available, due to lack of trained personnel, equipment, and infrastructure [13,18]. Additionally, provision of early ultrasound is reliant on timely initiation into care. In Uganda, only 60% of women received the recommended four antenatal care visits and the median gestational age at entry was 4.7 months in 2016 [19].

Therefore, in settings where antenatal ultrasound is not universal and where antenatal care coverage is inadequate, ultrasound use when a woman first presents to the maternity ward for labour may be beneficial. This time of assessment, known as labour triage, is a critical opportunity to identify conditions that have evolved during pregnancy, or that were missed during antenatal care, so that these cases can be prioritized if needed [20]. Efforts to ensure quality of care during the intrapartum period, beginning with entry into care at triage, is a key challenge facing LMICs like Uganda, where maternal and neonatal mortality rates remain high [21,22].

With the aim of improving the identification of six high-risk obstetric conditions at labour triage, the Preterm Birth Initiative East Africa conducted two parallel studies introducing a clinical assessment checklist and limited obstetric ultrasound at a district referral hospital [23] and three primary health centers [24] in eastern Uganda. Nested within these two studies, we ascertained patients’ perceptions of care upon receiving these interventions upon arrival to the labour ward, as well as providers’ experience with triage ultrasound. We specifically explored how introduction of point-of-care ultrasound in the maternity wards influenced perceptions of patient satisfaction, provider confidence, patient-provider communication, and work flow processes.

## Methods

### Study design

This mixed-methods study was nested within two studies that were conducted at a district hospital (DH) and three health centers (HCs) in eastern Uganda. These studies (hereafter, termed intervention studies) used a phased approach to evaluate the introduction of a clinical assessment checklist and/or limited obstetric ultrasound at labour triage on improving identification of six selected high-risk obstetric conditions prior to birth (multiple gestation, malpresentation, oligohydramnios, placenta previa, gestational age<37 weeks, and foetal heart rate abnormalities) [23,24]. Briefly, Phase 1 introduced an intake log to register all women who presented at the maternity ward, and an outcome form for baseline assessment; Phase 2 introduced a triage checklist that guided clinical assessment and decision-making at labour triage, and also provided support for patient transport in case of a referral from the HC to the DH; Phase 3 introduced both the checklist and midwife-administered limited obstetric ultrasound at labour triage. We hypothesized that these interventions–all of which were administered by facility midwives—would improve standardized documentation and intake processes at triage and subsequent clinical decision-making for a subset of obstetric conditions. Our a priori hypotheses for these intervention studies were that there would be differences in correct identification of obstetric conditions upon introduction of Phase 2 interventions compared to Phase 1, as well as Phase 3 interventions compared to Phase 1.

For this nested study that focused on women’s and providers’ perceptions, study research nurses administered a questionnaire to a sub-sample of participants from the intervention studies across the three phases. To understand provider perceptions around introduction of ultrasound at triage, we conducted key informant interviews (KIIs) among a subset of midwives trained in study procedures. While we believed women’s and providers’ experiences would be positive based on other published antenatal ultrasound studies [8–16], we recognized the labour triage setting would be a critical influencing factor.

### Study setting

This study was conducted at a DH, two level IV HC and one HC level III in Busoga region, eastern Uganda. The average monthly delivery volumes were approximately 640 at the DH and 60–75 deliveries at each HC. Typically, each DH shift is covered by approximately four midwives, one obstetrician/gynaecologist and one medical officer, while each HC shift is staffed by one midwife in the labour room and another servicing antenatal care. The catchment population is estimated at 20,000 for the HC III, 100,000 at HCIV, and over 2,000,000 for the DH. The three HCs are located approximately 10 to 40km away from the DH, and provide 24‐hour delivery services without caesarean delivery capacity. Prior to these studies, no ultrasound services were available at HCs, while at the DH it was solely available in the outpatient department where women were required to wait in queue and pay a fee.

### Study participants and recruitment

As part of the larger intervention studies, research nurses obtained consent from eligible women upon presentation to the maternity ward. Women who participated in the intervention study were then approached by a research nurse before they were discharged to ask if they would participate in a short questionnaire measuring her perceptions of care provided at the facility. If consent was obtained, a study research nurse administered the questionnaire. Using a confidence interval of 95% and a minimum power of 80%, we needed to survey at least 292 women per phase to understand their perception of the quality of care they received during labour (i.e., 292 per phase at the DH; 292 per phase across the three HCs).

As summarized in Table 1, a total of 815 women with linked intake data completed the survey– 216 (26.5%), 278 (34.1%) and 321 (39.4%) across the phases from the DH. A total of 731 women participated in the HC-level questionnaires, with 290 (39.7%), 332 (45.4%) and 109 (14.9%) across the three phases. Intended sample size was not reached in some phases given missingness or inability to link intake or outcome data. For HC Phase 3 specifically, lower enrolment rates for the larger intervention study influenced sample size for questionnaire completion [24].

**Table 1:** Recruitment of questionnaire participants across phases.

Phase	DISTRICT HOSPITAL RESPONDENTS		HEALTH CENTER RESPONDENTS	
	n	N_1_	N	N_2_
1	216	1234	290	1013
2	278	1219	332	870
3	321	1412	109	388
Totals	815	3865	731	2271

n = number of questionnaire respondents; N_1_ = number of women enrolled in the DH intervention study; N_2_ = number of women enrolled in the HC intervention study.

To ascertain provider perceptions of ultrasound, a qualitative researcher independent of the intervention studies conducted 12 KIIs with providers at the DH and HCs. At each of the three HCs, KIIs were conducted with 2 midwives who were trained as part of the ultrasound study for a total of 6 midwives. At the DH, four ultrasound-trained midwives were interviewed, two of whom were additionally trained as Master Trainers. These Master Trainers provided mentorship to the HC trainees [25], so it was an invaluable benefit to hear if or how their role as trainer influenced their perspectives.

### Data collection

Study research nurses collected women’s questionnaire data on paper-based forms. A 5-point Likert scale (e.g., excellent, very good, good, poor, very poor) was used to query topics related to patient perceptions of quality of care provided at triage, as well as experience of ultrasound scan (Phase 3 only). Free text responses related to reasons of perceived quality were coded into categories. This questionnaire (included in Supplemental Information) was not validated prior to use; however, during a 4-day training period before the study initiation, each question was read and discussed and to ensure clarity and correct interpretation. It was then pilot tested among women at the DH. Responses were transferred via tablet into Open Data Kit (ODK) and then to a secure server. All devices used for data entry (laptops or tablets) were encrypted and password protected.

All KIIs were conducted by a qualitative researcher, not part of the study team. The interview guide (Supplemental Information) was pilot tested on 2 midwives to determine appropriateness of the tool in terms of length, word choice, clarity of questions and flow. KIIs were audio recorded and a designated notetaker was present in all interviews. The audio recordings were transcribed verbatim and augmented with the use of the notes captured by the notetaker. All interviews were conducted in English. Interviews lasted between 45 minutes and one hour. Participants’ names were not recorded; rather, a participant ID number was assigned to ensure anonymity across all interviewees. Prior to the KIIs, informed written consent was obtained.

### Data analysis

For the quantitative data, all individual-level data from the questionnaire, intake log and outcome forms were linked through an individual study identification number and inpatient number. Bivariate analyses of women’s sociodemographic and reproductive health characteristics were conducted for categorical data using chi-square tests or Fisher’s exact statistics. Likert data related to patient perceptions of care were converted to dichotomous outcome variables: quality of care perceived as excellent or very good = 1, all other answers = 0; very satisfied or satisfied with care at triage = 1, all other answers = 0; overall facility recommendation rating very likely or likely = 1, all other answers = 0. All other answers included negative, neutral and missing responses. Chi-square tests or Fisher’s exact statistics were used to analyse data by phase. Where possible, logistic regression with adjustment for maternal age, education level, fuel source, and attendance to 4 antenatal care visits were conducted. Significance was considered at a p-value of less than 0.05. Given the sampling methods and differences in sample size across the phases, sampling weights were included in the analysis (S1 Table, Supplemental information). Patient-reported reasons for perceived care and perceptions regarding ultrasound were analysed descriptively.

For the provider KIIs, transcripts were reviewed by two readers. Codes were grouped into categories and then themes and subthemes were further identified and discussed. Themes were identified and coded in matrix tables using Excel.

### Ethical considerations

All study participants–women and providers—provided written informed consent. Participants were able to opt-out of the questionnaire or interview at any time, or not respond to certain questions if desired. Ethical approval for this study was obtained from the Institutional Review Board at the University of California San Francisco IRB (#17–23310) and the Higher Degrees, Research and Ethics Committee at Makerere University in Uganda (#515).

## Results

### Women’s perceptions

Characteristics of women stratified by DH and HC are shown in Table 2. The majority of mothers were between 20 and 35 years old. Participants’ education level, with higher numbers completing some university in Phase 3 compared to Phases 1 and 2, was different across phases. The primary source of cooking fuel (which served as an unvalidated proxy for socioeconomic status [26]) reported by phase was charcoal for DH (63.0%, 48.9%, 71.3%) and firewood for the HCs (76.9%, 93.7%, 87.2%) respectively. A significant difference among DH participants was observed for completion of four or more antenatal care visits by phase (35.6%, 37.8%, 75.8%), though this trend was not observed at the HC-level. As expected, the use of ultrasound during the current pregnancy was highest among participants recruited in Phase 3.

**Table 2:** Women’s sociodemographic and reproduction health characteristics across phases.

		DISTRICT HOSPITAL RESPONDENTS				HEALTH CENTER RESPONDENTS			
		Phase 1 (n = 216)	Phase 2 (n = 278)	Phase 3 (n = 321)	pvalue	Phase 1 (n = 290)	Phase 2 (n = 332)	Phase 3 (n = 109)	pvalue
Mother’s age (years)	<20	20.4%	15.8%	21.5%	0.438	19.7%	23.2%	22.9%	0.217
	20-<35	72.7%	75.2%	71.0%		75.1%	68.1%	72.5%	
	> = 35	6.9%	9.0%	7.5%		5.2%	8.7%	4.6%	
Education	None	0.5%	0.0%	0.6%	0.002^a^	0.0%	1.2%	0.0%	<0.0001^a^
	Some or completed Primary	49.8%	51.1%	42.9%		69.2%	76.5%	64.8%	
	Some or completed Secondary	45.9%	48.2%	49.8%		29.8%	21.4%	26.9%	
	Some or completed University	3.8%	0.7%	6.6%		1.0%	0.9%	8.3%	
Source of cooking fuel	Charcoal	63.0%	48.9%	71.3%	<0.0001 ^a^	23.1%	6.3%	12.8%	<0.0001 ^a^
	Gas, electric or paraffin	0.5%	1.4%	2.8%		0	0	0	
	Firewood	36.6%	49.6%	25.9%		76.9%	93.7%	87.2%	
Attended > = 4 antenatal care visits	No	64.4%	62.8%	24.2%	<0.0001	45.2%	49.7%	45.3%	0.496
	Yes	35.6%	37.2%	75.8%		54.8%	50.3%	54.7%	
US scan current pregnancy	no	90.3%	94.2%	0.3%	<0.0001	97.9%	99.7%	5.5%	<0.0001
	yes	9.7%	5.8%	99.7%		2.1%	0.3%	94.5%	

^a^Fisher’s exact test.

In weighted analyses (Table 3), DH participants’ perceived quality of care at the time of triage trended in the positive direction (i.e., from less to more satisfied) between Phase 2 and Phase 1, between Phase 3 and Phase 1, and between Phase 3 and Phase 2. However, satisfaction of care and overall recommendation rating was higher in Phase 2 relative to Phase 1 and to Phase 3, but was not statistically different between Phase 3 and Phase 1. At the HC-level, all measures increased in Phase 3 relative to both Phase 1 and Phase 2. Comparing Phase 2 to Phase 1, quality of care at the time of triage trended in the positive direction, but satisfaction and overall facility recommendation trended in the opposite direction, with less women expressing improvements.

**Table 3:** Women’s perceptions of care*.

**DISTRICT HOSPITAL RESPONDENTS**							
		Phase 1 (n = 216)	Phase 2 (n = 278)	Phase 3 (n = 321)	Weighted pvalue		
	Likert rating	%	%	%	Ph 2 vs. 1	Ph 3 vs. 1	Ph 3 vs 2
Rating of quality of care at triage	Else	86.6%	43.9%	29.6%	<0.001	<0.001	<0.001
	excellent or very good	13.4%	56.1%	70.4%			
Satisfaction of care at triage	Else	8.8%	2.9%	6.9%	<0.001	0.060	<0.001
	very satisfied or satisfied	91.2%	97.1%	93.1%			
Overall facility recommendation rating	Else	5.1%	1.4%	5.3%	<0.001	0.812	<0.001
	very likely or likely	94.9%	98.6%	94.7%			
**HEALTH CENTER RESPONDENTS**							
		Phase 1 (n = 290)	Phase 2 (n = 332)	Phase 3 (n = 109)	Weighted pvalue		
	Likert rating	%	%	%	Ph 1 vs. 2	Ph 1 vs. 3	Ph 2 vs 3
Rating of quality of care at triage	else	48.3%	30.4%	15.6%	<0.001	<0.001	<0.001
	excellent or very good	51.7%	69.6%	84.4%			
Satisfaction of care at triage	else	9.3%	13.0%	1.8%	0.010	<0.001	<0.001
	very satisfied or satisfied	90.7%	87.0%	98.2%			
Overall facility recommendation rating	else	5.9%	9.6%	0.0%	0.002	<0.001	<0.001
	very likely or likely	94.1%	90.4%	100.0%			

*data are weighted to account for sampling design.

It is worth noting that while satisfaction of care and recommendation measures had relatively positive responses during Phase 1 (>90%), ratings around perceived quality of care at triage was much lower at both the DH and HCs (13.4% and 51.7%, respectively).

Adjustment for differences in sample characteristics and survey weighting across study phases did not change results seen at the DH-level, or quality of care at triage and satisfaction at the HC-level (Table 4). However, we observed wide confidence intervals and an inability to fit the model for HC recommendation rating.

**Table 4:** Logistic regression of women’s perceptions of care controlled for sampling weights and covariates.

		DISTRICT HOSPITAL RESPONDENTS				HEALTH CENTER RESPONDENTS			
		OR	95% C.I.		p-value	OR	95% C.I.		p-value
			Lower	Upper			Lower	Upper	
Quality of care at triage	*Phase number 1 (ref)*								
	Phase number 2	8.34	6.81	10.21	<0.001	2.04	1.67	2.50	<0.001
	Phase number 3	16.45	13.27	20.38	<0.001	4.41	3.19	6.09	<0.001
Satisfaction of care at triage	*Phase number 1 (ref)*								
	Phase number 2	3.20	2.16	4.74	<0.001	0.71	0.52	0.97	0.033
	Phase number 3	1.14	0.84	1.55	0.399	4.25	1.95	9.26	<0.001
Overall facility recommendation rating	*Phase number 1 (ref)*								
	Phase number 2	3.80	2.21	6.50	<0.001	0.62	0.43	0.90	0.110
	Phase number 3	0.91	0.63	1.32	0.623	-	-	-	-

Controlled for sampling weights and covariates: Maternal age, education level, cooking fuel, and attendance at 4 antenatal care visits.

Respondents were asked to give reasons for perceived quality of care. Among both DH and HC participants who rated perceived quality of care at triage as excellent or very good, the leading reasons for perceived quality of care included having a healthy/safe delivery and being attended to by a caring and kind midwife (Table 5). In Phase 3, more women at both the DH and HCs stated that their perceived quality of care was due to having a knowledgeable and experienced midwife, compared to Phases 1 and 2 (Table 5).

**Table 5:** Reasons given for perceived care among participants who rated perceived quality of care at triage as excellent or very good.

	DISTRICT HOSPITAL RESPONDENTS			HEALTH CENTER RESPONDENTS		
	Phase 1 (n = 29)	Phase 2 (n = 156)	Phase 3 (n = 226)	Phase 1 (n = 150)	Phase 2 (n = 231)	Phase 3 (n = 92)
Healthy/safe delivery	72.4%	76.3%	73.9%	22.0%	21.2%	8.7%
Caring and kind midwife	10.3%	10.9%	15.5%	62.7%	55.8%	65.2%
Knowledgeable and experienced midwife	3.4%	3.8%	7.5%	7.3%	14.3%	26.1%
All other answers	13.8%	9.0%	3.1%	8.0%	8.7%	0.0%

n = number of women who rated perceived quality of care as excellent or good.

**Fig 1:**
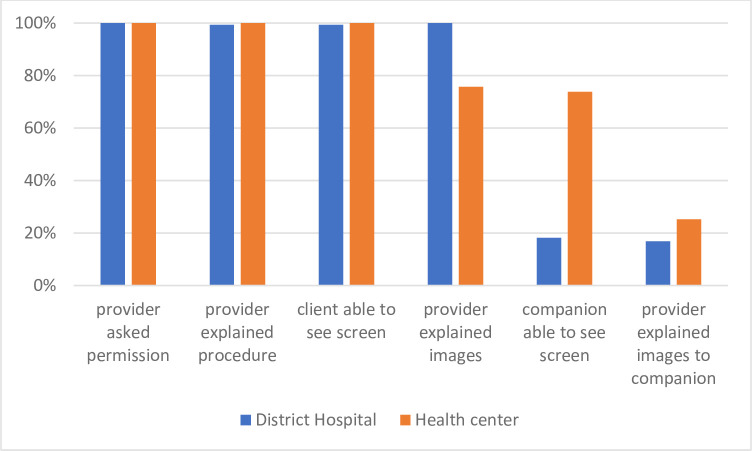
Patients’ perception on ultrasound at the different health facilities during Phase 3 (n = 103 health centers; n = 320 district hospital).

For Phase 3, we explored patient perceptions of ultrasound. Across both facility levels, women reported high rates of being asked permission, providers explained the procedure, and they were able to see the screen (Fig 1). However, differences were observed when asked if providers explained images to the patient (DH 100% vs HC 75.7%), as well as companions’ ability to see screen (DH 18.1% vs HC 73.8%).

While the majority of participants felt no risk and had no change in feelings after the scan, a significant proportion (11.1% and 14.4%) felt their feelings change after the scan. Among those who gave a reason for a change post-scan (n = 67), reasons included: (a) fear or worry, as mothers were scared of giving birth and caring for a preterm/small baby, big baby, or multiple gestation (17/67, 25.4%); (b) fear that the scanning procedure and gel used would harm the baby or client (36/67, 53.8%); (c) relief as they were worried about the baby’s wellbeing (9/67, 13.4%).

### Providers’ experience

The qualitative component of the study explored providers’ experience in ultrasound provision and implementation. Data are presented under four thematic areas: (1) workflow integration; (2) impact on clinical processes; (3) perceived patient response to ultrasound; and (4) implementation barriers.

#### Thematic area 1: Workflow integration

Key informants were asked about how they were able to integrate ultrasound into their existing workflow routine. All 12 respondents mentioned that they did the scanning immediately after taking the mother’s history and conducting a physical examination. In this regard, they found it easy to integrate these services as a continuation of the care they provide on admission.

*“We have integrated it in this way that after the mother has come at the reception, you assess, you take history, then vitals, after you take her for physical examination, you examine her abdomen, then you assess whether she is in labour or not. If she is in labour and has consented, you begin filling the checklist up to the part for examination and then you scan since we are using the same room for both services. So this has helped us to integrate immediately. Then after scanning you go ahead and fill the rest of the behind part of the checklist, then after I continue with my management according to the results from the scan.” (KI-DH)*

A majority (7/12) of the participants reported that the introduction of triage ultrasound increased the amount of time spent with each mother. This included time to scan, interpret results, and counsel the mother. High patient volume was an issue expressed specifically by DH midwives.

*“The time spent on a mother increased because previously, we used to just examine them, admit her in the file and then continue monitoring. But with the introduction of the scan, after examining, you scan her and then admit her in the file. So generally, the time lag spent on one patient increased yet you may have very many patients waiting.” (KI-DH)*

*“Well, the time increased somehow but not in a bad way, the ultrasound activity would take like 10 to 20 minutes which was not a very big time difference. Before, I would just examine, listen to the foetal heart, presentation and so forth and end there, this would take very little time but currently I will need an extra time to further take the mother to the scan.” (KI-HC)*

A few key informants, specifically at the HC-level, also reported that the introduction of ultrasound interfered with their work responsibilities. It increased the waiting time for other patients in other departments and affected efficiency of these services.

*“We are few health workers at the facility, so sometimes you may be alone on duty and have a long queue waiting for ANC for example on clinic days, on MBCP (Mother Baby Care Point) days, and with the ultrasound scan, you can take like 45 minutes on a mother including the scan, explanations about the results, answering her questions and in case of any issues, referring her.” (KI-HC).*

As opposed to the above reports of workflow disruption, some (5/12) participants reported a positive impact of ultrasound on workload, particularly with regard to offering more patient-centred care. Ultrasound helped providers identify certain conditions faster, facilitating clinical decision-making. This, in turn, was considered more efficient for the mother.

*“To me, my work load was not affected because it quickened my services to the mother. Before the introduction of the scan, you could get a mother and suspect fetal heart rate, not getting the presentation, hence sending them to town or even OPD [out-patient department] and this was time wasting, sometimes the mother could be draining and you make her move around. So the time reduced because we are offering all services from here.” (DH-KI).*

#### Thematic area 2: Impact on clinical processes

All respondents reported that ultrasound helped reinforce their clinical decision-making. They felt that physical examinations tended to give inaccurate results, whereas ultrasound was less prone to error. They felt ultrasound helped build confidence in assessment and decision-making.

*“Yes, like one mother came and she presented when she was okay, no complaint of bleeding and nothing. When I examined her, I also felt she had no problem but when I scanned her, she had a previa. The placenta was low lying and obviously this had to change my management plan. I referred her to the doctor who opted for another scan and the results were the same and they operated on her.” (KI-DH)*.

*“Yes, like for several times, it has helped me to diagnose babies with bad heart rate because when you are listening with just a fetoscope, you may listen that the heart is fast but it is very difficult to get the right fetal heart, but with a scan, it has helped me to differentiate that this is a fetal distress … making me take quick intervention.” (KI-DH).*

A few HC-level key informants also reported that the ultrasound scan facilitated decision to refer.

*“Yes, it has changed in that before, we could keep mothers with complications who would not manage to push the babies but with ultrasound, we have managed to identify them as early as possible and we refer such mothers.” (KI-HC).*

#### Thematic area 3: Perceived patient response to ultrasound

Key informants were asked if they felt women liked getting an ultrasound scan and most responded in affirmative. They reported that women’s interest in the ultrasound was evidenced by excessive complaints from mothers that were not scanned due to eligibility reasons (e.g., less than 28 weeks of gestation or women attending routine antenatal services), as well as few women who refused to consent.

*“Also, there are very few cases of mothers who are not willing to receive the scan, so even those very few, they do not receive a scan, they can be like 1 in 50 mothers.” (KI-HC).*

Providers also stated that patients appreciated ultrasound because they felt involved in their care.

*“Even the mothers are very comfortable, when you take her to the scan, she will feel safe since we tell them we shall see the condition of the baby inside the stomach. The mother will feel the care too.” (KI-HC)*.

*“They would request that they wanted to also see the baby in the stomach and we would show them how the heart is pumping which leaves them excited.” (KI-DH).*

Midwives reported that ultrasound acceptability was influenced by the fact that it was offered for free.

*“Before we used to send them to town to have a scan done in those private health facilities. So when we introduced the scan to them, they actually thought they were going to pay, we explained to them that it was for free of charge and everybody could just follow you up to have a scan done.” (KI-HC).*

However, some respondents noted that mothers did not want the scan and refused to consent for a myriad of reasons.

*“Some patients who have negative attitude towards it, they had perception that if you go for scan, it reduces on the life span due to that power that runs through and they would oppose the idea.” (KI-HC)*

*“There are also some mothers who would come when they are approaching second stage of labour, when you tell her to first come for the scan, she refuses to consent because she is only interested in pushing hence you leave her.” (KI-DH)*

*“Sometimes, you can talk to them and they refuse. The refusal is because some mothers fear their men” (KI-DH).*

#### Thematic area 4: Implementation barriers

The study was interested in understanding health systems factors that inhibited use of ultrasound at labour triage. First, an overwhelming majority of key informants reported that electricity supply at their facilities was very unreliable, with regular outages which made it hard to provide the ultrasound scan services consistently.

*“The main barrier is power… Since we are in a village, when we get any power outage, it can take some time without being reconnected compared to the situation in town. Once the transformer gets a problem, we can even spend two weeks without electricity.” (KI-HC)*.

*“The main barrier is power outages and when the electricity goes off, we do not have a standby generator implying that during that period, we would not scan the mothers.” (KI-DH).*

Second, it was reported that because not all midwives were trained in study procedures, including ultrasound, the few who were trained could not cover all shifts.

*“Since we were only two people doing the ultrasound scan, you would find in case one of us is on duty especially night duty, she can become tired because mothers are always many and give birth in the night. So because of a busy schedule, you may find when she opts to scan a few and do other activities that are also important.” (KI-HC).*

Lastly, it was reported by most respondents that too much workload left them with no time to provide ultrasound services to all mothers. In such situations, multi-tasking was difficult and eligible women were not scanned.

*“At times it is workload. You may have very many mothers and the staff are few, you cannot scan all the mothers even if they are eligible since you also have to do other pending activities.” (KI-DH)*.

*“The barrier would come in case we got many mothers in maternity who were in labour and others delivering at the same time. We would be very busy and would not concentrate on scanning all the eligible mothers. We would then miss some of them since they deliver before being scanned.” (KI-HC).*

## Discussion

This mixed methods study explored perceptions of two triage interventions at a DH and three HCs in Eastern Uganda from both women and providers’ perspectives. First, we found that a standardized checklist used at triage to assess for obstetric conditions greatly improved the rating of quality of care at triage at both the DH and HCs. Checklists, such as the Safe Childbirth Checklist, have been shown to increase adherence to evidence-based practices [27], as well as improve perceptions around respectful maternity care and perceived quality of care [28]. Furthermore, introduction of a more formalized obstetric triage system has been shown to improve initial diagnostic assessments and reduce patient delays [29]. We hypothesize that the introduction of the triage checklist not only standardized initial assessments, but also increased interaction time between provider and patient.

Second, upon introduction of ultrasound in Phase 3, women’s perceived quality of care at triage increased relative to both Phase 1 and Phase 2 at both the DH and HCs. We found that among DH women who rated perceived quality of care as excellent or good, the perception that midwives were knowledgeable and experienced, or caring and kind, was more commonly cited in Phase 3. At the HC-level, women cited knowledge and experience as a key reason for their rating. This sentiment was echoed by providers, as they felt that ultrasound helped in making correct clinical management plans and facilitated decision making.

In contrast, however, satisfaction of care at triage and overall facility recommendation rating did not significantly differ upon introduction of ultrasound at the DH, but improved among HC respondents. Both of these measures were greater than 90% in Phase 1, with nominal changes at the DH and more pronounced improvements at the HCs. One potential explanation for this discrepancy is that the DH which is situated in a peri-urban environment has a much higher patient volume than the HCs. DH midwives expressed that ultrasound increased workload in the context of high patient volume and limited staff; in situations like this, midwives’ time at the patient bedside after administration of ultrasound was reduced, and may have contributed to the overall perception of poor satisfaction. This may point to why DH patients felt that triage quality improved, but overall services rendered post-admission did not. Another potential explanation for why satisfaction did not improve at the DH may be related to lack of social support during the scan. Our findings indicated that a considerable proportion of DH patients stated that their companions could not see images or that results were not conveyed to them. Anecdotally, the DH triage room was small with a long queue of patients awaiting assessment. Thus, there was limited opportunity to have a companion in the triage room during the scan due to complicated triage flow mechanisms and high midwife workload, which could have influenced satisfaction of care ratings.

We observed that the majority of participants viewed ultrasound as beneficial and expressed lack of fear of being scanned. It was offered as a free service and patient involvement during the ultrasound process enhanced acceptability of the service at both the HC and DH levels. Nonetheless, a considerable proportion of participants expressed fear and a change in feelings after the scan. Some patients stated that the ultrasound results influenced their anxiety level in a positive way after learning that their babies were well; however, in other instances, some expressed increased fear or anxiety when they learned of potential poor outcomes. These sentiments align with a previous study among rural pregnant women which described both fear and relief following a fetal ultrasound experience [14], and another study whereby introduction of ultrasound was found to sometimes cause psychological stress among pregnant women due to fear of detecting fetal anomalies [30]. Of note, a considerable proportion of participants in this study expressed fear and safety concerns related to the machine or scanning gel, but these issues were not consistently raised by the key informants. This suggests the need for improved communication with a standard explanation to alleviate fears. Similar findings for lack of communication between patients and health providers offering ultrasound in LMIC have been reported in other settings [13], with speculation that the technical aspect of performing an ultrasound impedes communication. One prior study also from rural Uganda showed that when the best practice of pairing medical procedures with patient education and proper communication is achieved, clinicians can improve patient perception of quality of care [22]. As our midwives were relatively novice users of ultrasound, the ability to multi-task technically (e.g., perform the ultrasound, clinically interpret the images and formulate a new patient delivery plan) while perceiving perhaps subtle patient cues of anxiety or desire for further explanation may have been missed. Use of written resources have proved to be beneficial in providing parental support in developed countries [31], though there is need to further explore the potential of integrating this kind of additional support and counselling in LMICs.

While our results suggest that triage ultrasound is seen as beneficial by the majority of mothers and as an important tool in decision making among providers, we also uncovered important system-level barriers to ultrasound implementation. Factors such as workload, inadequate staffing and inconsistent power have been noted in prior literature [12,32,33]; however, by exploring these issues at both a DH and HCs, we have uncovered barriers and facilitators that affect ultrasound implementation at two different levels of government facilities. Addressing these key issues will be critical in enhancing quality of maternity care, and have policy implications regarding resource allocation [34].

### Study limitations

The study has several limitations. First, our study participants differed in some characteristics across phases and intended HC sample size was not reached in Phase 3, though we addressed these issues by weighting and adjusting for covariates. Second, differences in Phase 3 demographics (e.g., higher education level, attendance to the recommended four antenatal care visits) could indicate different health-seeking behaviours among these women, thus potentially introducing some bias toward acceptability of ultrasound. Third, due to study resources, we did not conduct in-depth interviews with women. This would have offered additional, more nuanced, insight that was not necessarily captured in a Likert-based survey. Fourth, we employed research nurses to consent women from the intervention study to participate in the questionnaire. Although all clinical activities associated with the intervention studies, including completion of the intake form, checklist, and ultrasound procedure were conducted by study-trained midwives, it is possible this introduced some bias–for example, women may have been more likely to consent or answer questions positively as nurses may have been perceived as tied to clinical care. Lastly, while our protocol included midwives to explain ultrasound safety and consent in a standardized manner, there were differences in adherence to this between our sites and among midwives. Thus, a written script with standardized communication may have improved compliance, particularly in busier clinical environments. Alternatively, a dedicated midwife to conduct scans each shift may have introduced more standardized practice, and would allow time for both pre- and post-procedure counselling (e.g., to explain the procedure, emphasize safety, demonstrate equipment, and answer questions).

## Conclusion

Obstetric ultrasound at the point of labour triage can be useful in contexts of inadequate antenatal care. However, its successful introduction is influenced by health system and user challenges, as well as perceptions among pregnant women. In consideration of these barriers and facilitators to implementation, comprehensive clinical training must also emphasize optimization of midwives’ knowledge, skills and confidence to ensure technical expertise is paired with respectful communication and counselling to alleviate patient fears of ultrasound.

## Supporting information

S1 Table Sampling weight assignment. (DOCX)

S1 File Patient questionnaire HC level. (DOCX)

S2 File Patient questionnaire DH level. (DOCX)

S3 File Interview guide tool. (DOCX)
