# Supplementary material for: Ultrasound at labour triage in eastern Uganda: A mixed methods study of patient perceptions of care and providers’ implementation experience
Source: PLoS One. 2021 Nov 12;16(11):e0259770. doi: 10.1371/journal.pone.0259770 (PMC8589172; doi:10.1371/journal.pone.0259770)
Supplement: S1 Table — (DOCX) [file pone.0259770.s001.docx]

**Table S1. Sampling weight assignment.**

|  | Questionnaire respondents | Women in intervention study | Proportion: respondents/ population | Sampling weight |
| --- | --- | --- | --- | --- |
| DH Phase 1 | 216 | 1234 | 0.175 | 5.713 |
| DH Phase 2 | 278 | 1219 | 0.228 | 4.385 |
| DH Phase 3 | 321 | 1412 | 0.227 | 4.399 |
| DH Total | 815 | 3865 |  |  |
|  |  |  |  |  |
| HC Phase 1 | 290 | 1013 | 0.286 | 3.493 |
| HC Phase 2 | 332 | 870 | 0.382 | 2.620 |
| HC Phase 3 | 109 | 388 | 0.281 | 3.560 |
| HC Total | 731 | 2271 |  |  |
| Weights calculated by taking the inverse of the sampling fraction. | | | | |
