# Supplementary material for: Ultrasound at labour triage in eastern Uganda: A mixed methods study of patient perceptions of care and providers’ implementation experience
Source: PLoS One. 2021 Nov 12;16(11):e0259770. doi: 10.1371/journal.pone.0259770 (PMC8589172; doi:10.1371/journal.pone.0259770)
Supplement: S1 File — (DOCX) [file pone.0259770.s002.docx]

| **Facility name** | **Patient Questionnaire (administered by data collector)** | | | | |
| --- | --- | --- | --- | --- | --- |
| Mother’s Name: | | | Today’s Date: | | |
| Study ID #: | | | Date mother gave birth: | | |
| IP #: | | | Data collector’s name: | | |
| **Question (Conditional logic)** | | | **English version** | | **Answer options** |
| 1. Wafuna okukeberebwa kw’akatiivi bwewatuuka eiganga kuwoodi ey’abali okulumwa? Okukeberwa kw’akativi kitegeeza omusawo okukozesa ekyuuma okubona munda ng’akozesa ebizigo n’ekyuuma ekiriku endabirwaamu | | | **Did you receive an ultrasound examination when you arrived at the Iganga Hospital labour ward? An ultrasound is when a provider uses a machine to see inside your body, using gel and a machine with a screen.**  **If no, skip to question 12** | | □ Yii (Yes)  □ Bbe (No) |
| 1a.Ogwo n’ogwaali omulundigwo ogusooka ku katiivi? | | | **Was that your first ultrasound?** | | □ Yii (Yes)  □ Bbe (No) |
| 2. Omusawowo yakusaba olukusa ng’akaali kukola mutendera gwakatiivi? | | | **Did your care provider ask for your permission for the ultrasound procedure before doing it?** | | □ Yii (Yes)  □ Bbe (No) |
| 3. Omusawo yainhonola omutendera nga akaali kugukola? | | | **Did the provider explain the ultrasound procedure before doing it?** | | □ Yii (Yes)  □ Bbe (No) |
| 4. Wabona ku ndabirwaamu y’akatiivi? | | | **Did you see the ultrasound screen?** | | □ Yii (Yes)  □ Bbe (No) |
| 5. Eyakuwerekeraku yabonaku kundabirwamu y’akatiivi? | | | **Did the provider explain the ultrasound screen images to your companion?** | | □ Yii (Yes)  □ Bbe (No) |
| 6. Omusawo yakwinhonola ebifaananie ebyaali kundabirwaamu y’akatiivi? | | | **Did the provider explain the ultrasound screen images to you?** | | □ Yii (Yes)  □ Bbe (No) |
| 7. Omusawo yainhonola oyo eyakuwerekeraku ebifaananie ebyaali kundabirwamu y’akatiivi? | | | **Did the provider explain the ultrasound screen images to your companion?** | | □ Yii (Yes)  □ Bbe (No) |
| 8.Kiki omusawo kyeyakukoba kubyaava mu katiivi? | | | **What did the provider tell you about the results of the ultrasound?** | | □ Bulikintukyaalibukalamu  **Everything was normal**  □ Eriyoekitalikikalamu  **Something was abnormal**  □ Tiidhi **I don’t know** |
| 8a. (Singa “eriyo ekyaali ekikyaamu mu 8) Omusawo yakukobaki? | | | **(If “Something was abnormal in 9) What did the provider tell you?** | | Free text |
| 9. Wawulira otya kukukeberebwa kw’akatiivi? | | | **How did you feel about the ultrasound exam?** | | Free text |
| 10. Wawulira nga waligho obuzibu bwoonabwoona obwekuusa ku kukeberebwa kw’akatiivi? | | | **Did you feel there was any risk associated with undergoing an ultrasound exam?** | | □ Yii (Yes)  □ Bbe (No) |
| 11. okwenda kwewalina eri omwanawo kwakyuuka muuku ng’omaze okubona akatiivi? | | | **Did your feelings toward your baby change after seeing the ultrasound?** | | □ Yii (Yes)  □ Bbe (No) |
| 11a. Bwekibayyi, nkusaba oinhonole engeri okubona akatiivi yekwakosa okwenda kwewalinakwo eri omwaanawo. | | | **If Yes, please explain how seeing the ultrasound affect your feelings toward your baby** | | Free text |
| 12. Idaalaki kwota omutindo gwendabirira yewafuna okuva eri omusawo bwewatuuka kuwoodi ey’abakyala abalumwa? | | | **How would you rate the quality of care you received from the provider when you arrived at the labour ward?** | | □ 1 Gwa wansi inho **Very poor**  □ 2 Gwawansi **Poor**  □ 3 Mulungi **Good**  □ 4 Mulungi inho **Very good**  □ 5 Muswiifu **Excellent** |
| 13. Oli musiimu kwagagha olw’endabirira yewafuna okuva eri omusawo bwewatuuka ku woodi y’abakyala abalumwa? | | | **How satisfied are you with the care you received from the provider when you arrived at the labour ward?** | | □ 1 Tiri musanhufu ghaire **Not satisfied at all**  □ 2 Tiri musanhufu **Not very satisfied**  □ 3 ndiagho ntyo **Neutral**  □ 4 Musiimu **Satisfied**  □ 5 Musiimu inho **Very satisfied** |
| 14. Nkusaba oinhonole byoizeemu | | | **Please explain your answers.** | | Free text |
| *(Data collector: If the mother had an ultrasound but didn’t say anything about the ultrasound in question 14a, ask)*  14a. Okugya kukatiivi ku woodi ey’abakyala abalumwa kyakosa engeri y’ogerangeraniamu omutindo gw’endabirira? | | | **Did receiving an ultrasound at the labour ward affect how you rate the quality of care?** | | □ Yii (Yes)  □ Bbe (No) |
| 14b Bwekiba yyi ku 14a, nkusaba oinhonole engeri oba lwaki okugya kukatiivi kyakosa engeri y’ogerangerania omutindo gw’endabirira? | | | **If yes to 14a, please explain how or why receiving an ultrasound affected how you rate the quality of care** | | Free text. |
| 15. Osobola kugerangerania otya omutindo gw’endabirira yewafuna okusinziira ku ibbanga lyoonalyoona lyewamala mu irwaliro?? | | | **Considering your entire stay in the facility, how would you rate the quality of care you received?** | | □ 1 Gwa wansi inho Very poor  □ 2 Gwawansi Poor  □ 3 Mulungi Good  □ 4 Mulungi inho Very good  □ 5 Muswiifu Excellent |
| 16. Eirwaliro lino oyinza kulisemba kyaagagha eri mukwanogwo oba ab’omumakaago?? | | | **How likely is it that you would recommend this facility to your family or friend?** | | □ 1 Tisobolerairala **Not likely at all**  □ 2 Tisobola **Not very likely**  □ 3 Ninza obutasobola obaokusobola (tiidhi) **Neither likely nor unlikely (I don’t know)**  □ 4 Niinza **Likely**  □ 5 niinza inho **Extremely likely** |
| *If mother did not have an ultrasound, end here.* | | | | | |
| 17 Ebidhuubo ebindi ebigema kundabirirayo oba akatiivi? | | | **Any other thoughts regarding your care or the ultrasound?** | | Free text |
| *End* | | | | | |
