# Supplementary material for: Ultrasound at labour triage in eastern Uganda: A mixed methods study of patient perceptions of care and providers’ implementation experience
Source: PLoS One. 2021 Nov 12;16(11):e0259770. doi: 10.1371/journal.pone.0259770 (PMC8589172; doi:10.1371/journal.pone.0259770)
Supplement: S2 File — (DOCX) [file pone.0259770.s003.docx]

| Iganga Hospital | **Patient Questionnaire (administered by data collector)** | |
| --- | --- | --- |
| Mother’s Name: | | Today’s Date: |
| Study ID #: | | Date mother gave birth: |
| IP #: | | Data collector’s name: |

| **Question (Conditional logic)** | **English version** | **Answer options** | |
| --- | --- | --- | --- |
| 1. Wasindikibwa wano okuva: | **Were you referred here from:** | □ Nsinze HC  □ Namungalwe HC  □ Bugono HC  □ None of these | |
| 1a. (If one of study sites) Baakukebereraku ku katiivi kuirwaliro eryo? Okukeberwa kw’akatiivi n’omusawo okukozesa ekyuuma okubona munda ng’akozesa ebizigo n’ekyuuma ekiriku endabirwaamu. | **Did you receive an ultrasound examination at that facility?**  **An ultrasound is when a provider uses a machine to see inside your body, using gel and a machine with a screen.** | □ Yii (Yes)  □ Bbe (No) | |
| 1b. (If yes to 1a) Ogwo n’ogwaali omulundi gwo ogusooka ku katiivi? | **Was that your first ultrasound?** | □ Yii (Yes)  □ Bbe (No) | |
| 1c. (If yes to 1a) Omusawo yakukobaki ku byaava mu kukebera kw’akativi? | **What did the provider tell you about the results of the ultrasound?** | □ Bulikintukyaalibukalamu  **Everything was normal**  □ Eriyoekitalikikalamu  **Something was abnormal**  □ Tiidhi **I don’t know** | |
| 1d. (If “something was abnormal”) Nkusaba oinhonole omusawo kyeyakukoba: | **Please explain what the provider told you:** | Free text | |
| 1e. (If yes to 1a) Ku kigero ky’e 1-5 ebyaava mu katiivi byakuyamba kwagagha mu kusalawo okwiidha mu irwaliro ly’e Iganga? | **On a scale of 1-5, how important was the ultrasound result in your decision to come to Iganga Hospital?** | □ Ti byaalibyamugasoghaire  **Not important at all**  □ Ti byamugaso  **Not very important**  □ Ezirakyebyankolaku  **It didn’t affect me**  □ Byalibyamugasomuuku  **Somewhat important**  □ Byamugasoinho  **Very important** | |
| 1. Wafuna okukeberebwa kw’akatiivi bwewatuuka eiganga ku woodi ey’abakyala abali kulumwa? Okukeberwa kw’akativi kitegeeza omusawo okukozesa ekyuuma okubona munda ng’akozesa ebizigo n’ekyuuma ekiriku endabirwamu. | **Did you receive an ultrasound examination when you arrived at the Iganga Hospital labour ward? An ultrasound is when a provider uses a machine to see inside your body, using gel and a machine with a screen**.  **If no, skip to question 13** | □ Yii (Yes)  □ Bbe (No) | |
| 2a. Oba yii, ogwo n’ogwaali omulundi gwo ogusooka kukatiivi? | **If Yes, was that your first ultrasound?** | □ Yii (Yes)  □ Bbe (No) | |
| 3. Omusawowo nga akaali kukola mutendera gwa katiivi, yakusaba olukusa? | **Did your care provider ask for your permission for the ultrasound procedure before doing it?** | □ Yii (Yes)  □ Bbe (No) | |
| 4.Omusawo yainhonola omutendera nga akaali kugukola? | **Did the provider explain the ultrasound procedure before doing it?** | □ Yii (Yes)  □ Bbe (No) | |
| 5. Wabona ku ndabirwaamu y’akatiivi? | **Did you see the ultrasound screen?** | □ Yii (Yes)  □ Bbe (No) | |
| 6. Eyakuwerekeraku yabonaku kundabirwamu y’akatiivi? | **Did your companion see the ultrasound screen?** | □ Yii (Yes)  □ Bbe (No) | |
| 7. Omusawo yakwinhonola ebifaananie ebyaali kundabirwaamu y’akatiivi? | **Did the provider explain the ultrasound screen images to you?** | □ Yii (Yes)  □ Bbe (No) | |
| 8. Omusawo yainhonola oyo eyakuwerekeraku ebifaananie ebyaali kundabirwamu y’akatiivi? | **Did the provider explain the ultrasound screen images to your companion?** | □ Yii (Yes)  □ Bbe (No) | |
| 9. Kiki omusawo kyeyakukoba kubyaava mu katiivi? | **What did the provider tell you about the results of the ultrasound?** | □Buli kintu kyaali bukalamu  **Everything was normal**  □ Eriyo ekitaali kikalamu  **Something was abnormal**  □ Tiidhi  **I don’t know** | |
| 9a. (Singa “eriyo ekyaali ekikyaamu mu 9) Omusawo yakukobaki? | **(If “Something was abnormal in 9) What did the provider tell you?** | Free text | |
| 10. Wawulira otya kukukeberebwa kw’akatiivi? | **How did you feel about the ultrasound exam?** | Free text | |
| 11. Wawulira nga waligho obuzibu bwoonabwoona obwekuusa ku kukeberebwa kw’akatiivi? | **Did you feel there was any risk associated with undergoing an ultrasound exam?** | □ Yii (Yes)  □ Bbe (No) | |
| 12. okwenda kwewalina eri omwanawo kwakyuuka muuku ng’omaze okubona akatiivi? | **Did your feelings toward your baby change after seeing the ultrasound?** | □ Yii (Yes)  □ Bbe (No) | |
| 12a. Bwekibayyi, nkusaba oinhonole engeri okubona akatiivi yekwakosa okwenda kwewalinakwo eri omwaanawo. | **If Yes, please explain how seeing the ultrasound affected your feelings toward your baby** | Free text. | |
| 13. Okusinziira ku ibbanga lyoonalyoona lyewamala mu irwaliro, ogeragerania otya omutindo gw’endabirira yewafuna bwewatuuka ku woodi y’abakyala abalumwa? | **How would you rate the quality of care you received from the provider when you arrived at the labour ward?** | □ 1 Gwa wansi inho Very poor  □ 2 Gwawansi Poor  □ 3 Mulungi Good  □ 4 Mulungi inho Very good  □ 5 Muswiifu Excellent | |
| 14. Oli musiimu kwagagha olw’endabirira yewafuna okuva eri omusawo bwewatuuka ku woodi y’abakyala abalumwa? | **How satisfied are you with the care you received from the provider when you arrived at the labour ward?** | □ 1 Tiri musanhufu ghaire **Not satisfied at all**  □ 2 Tiri musanhufu **Not very satisfied**  □ 3 ndiagho ntyo **Neutral**  □ 4 Musiimu **Satisfied**  □ 5 Musiimu inho **Very satisfied** | |
| 15. Nkusaba oinhonole byoizeemu | **Please explain your answers.** | Free text | |
| (Data collector: If the mother had an ultrasound but didn’t say anything about the ultrasound in question 15, ask)  15a. Okugya kukatiivi ku woodi ey’abakyala abalumwa kyakosa engeri y’ogerangeraniamu omutindo gw’endabirira? | **Did receiving an ultrasound at the labour ward affect how you rate the quality of care?** | □ Yii (Yes)  □ Bbe (No) | |
| 15b. Bwekibayyiku 15a, nkusaba oinhonole engeri oba lwaki okugya kukatiivi kyakosa engeri y’ogerangerania omutindo gw’endabirira? | **If yes to 15a,**  **please explain how or why receiving an ultrasound affected how you rate the quality of care** | Free text | |
| 16.Osobola kugerangerania otya omutindo gw’endabirira yewafuna okusinziira ku ibbanga lyoonalyoona lyewamala mu irwaliro? | **Considering your entire stay in the facility, how would you rate the quality of care you received?** | □ 1 Gwa wansi inho **Very poor**  □ 2 Gwawansi **Poor**  □ 3 Mulungi **Good**  □ 4 Mulungi inho **Very good**  □ 5 Muswiifu **Excellent** | |
| 17. Eirwaliro lino oyinza kulisemba kyaagagha eri mukwanogwo oba ab’omumakaago? | **How likely is it that you would recommend this facility to your family or friend?** | □ 1 Tisobolerairala **Not likely at all**  □ 2 Tisobola **Not very likely**  □ 3 Ninza obutasobola obaokusobola (tiidhi)  **Neither likely nor unlikely (I don’t know)**  □ 4 Niinza **Likely**  □ 5 niinza inho **Extremely likely** | |
| (If the mother did not have an ultrasound, end here.) | | | |
| 18. Ebidhuubo ebindi ebigema kundabirirayo oba akatiivi? | **Any other thoughts regarding your care or the ultrasound?** | Free text | |
| End | | |  |
