# Supplementary material for: Ultrasound at labour triage in eastern Uganda: A mixed methods study of patient perceptions of care and providers’ implementation experience
Source: PLoS One. 2021 Nov 12;16(11):e0259770. doi: 10.1371/journal.pone.0259770 (PMC8589172; doi:10.1371/journal.pone.0259770)
Supplement: S3 File — (DOCX) [file pone.0259770.s004.docx]

**Appendix V: PROVIDER INTERVIEW GUIDE**

***Impact of introducing a clinical assessment checklist and limited obstetric ultrasound during labour triage to improve the detection of six high-risk obstetric conditions in select health facilities in Busoga Region, Uganda: a pre-post intervention study***

Hello, my name is *[state your name].* Thank you for your willingness to take part in this interview. I am here today to learn about your experiences as a health care provider who received training in limited obstetric ultrasound as part of the study. This interview should take between 45 minutes and one hour.

We are hoping you feel comfortable with us audio-recording this interview and taking some notes. None of your answers will be shared with your supervisors or colleagues at work. What you say will be shared with the ultrasound study team, but your name and location of work will not be connected to your answers in order to maintain your confidentiality. We appreciate your honest answers and respect all your opinions and thoughts. You can choose not to answer any of our questions at any time.

Date of interview: ______/ _______/ ____________

Venue: _______________________________

Designation of Respondent: ____________________

Interviewer _______________________

Time started: ________________

Time ended: _________________

Key informant interview identifier: __ *(KII/designation/date/cumulative no of KII done)* __________________________

**Interviewer, note start time and begin audio-recording*

**Section 1: INTRODUCTION**

1. I’d like to get to know you a little. What is your position here and how long have you worked here?
2. What is your role in the ultrasound study?
3. Could you walk me through what a typical day looks like for you?

**Section 2: WORKFLOW INTEGRATION and IMPACT ON CLINICAL PROCESSES**

As you know, the purpose of the study was to determine if two interventions – a clinical checklist and limited obstetric ultrasound – can affect identification of high-risk conditions and subsequent clinical management. Today, we are interested in hearing your thoughts about these interventions.

1. How did you integrate the ultrasound scan into your normal workflow routine? *Probe for specific information on sequence of workflow, e.g. was ultrasound done after physical exam? after history?*
2. How do you think introduction of ultrasound affected your workload? *Probe for time per patient?*
3. Do you think every woman who came in received a scan? If not, why not? Which kind of mothers are most difficult to scan and why? *Probe for night vs. day shift challenges.*
4. Did ultrasound ever change your clinical plan? Can you provide an example?
5. In your opinion, which part of the study protocol influenced your patient care, either in a positive or negative way? Why*?*
6. Of the six complications you were taught to identify by ultrasound (fetal heart rate, multiples, fluid volume, placenta location, head location, gestational measures):
   1. What was the most difficult for you to measure or image?
   2. What was the easiest*?*
   3. What was the most valuable to you clinically?
7. Do you think providing ultrasound at triage is a useful tool and should be provided to all women in labor? Why or why not? *Probe - would you change anything in terms of workflow, complications to assess, specific sub-set of women to assess?*
8. What do you think was the added value, if any, of the ultrasound scan versus the triage checklist?
   1. For Iganga respondents only – Are you familiar with the modified Safe Childbirth Checklist (mSCC) that was introduced by the larger PTBi study (it had 5 pause points)? If yes, which checklist (the full mSCC or this study’s triage checklist) did you find more useful? Why?

**Section 3: PATIENT-PROVIDER RELATIONSHIP**

1. Do you think women liked getting an ultrasound scan? Do you have any patient stories you remember?
2. What were some common questions mothers would ask you during the scan?

**Section 4: ADEQUACY OF HEALTH SYSTEM INFRASTRUCTURE**

1. What were the main facilitators that enabled you to use ultrasound in your facility?
2. What were the main barriers to using the ultrasound consistently in your facility? *Probes: Electricity? Training? Time needed? Availability of gel/clean towels? Night vs. day shift challenges?*
3. Did you have any ultrasound equipment issues that arose and if so what (specifically for the MindRay machines, not SonoScape machines)? *Probes: Machine overheating, error measurements, battery life, charging time, keyboard/motherboard/probe problems?*
4. Did you have issues with machine security or access when you needed it? *Probes: Was it locked up? Did others want to use the machine for different areas and departments?*
5. What would make ultrasound scanning more convenient at labor triage and why?

**Section 5: PERCEPTIONS ABOUT ULTRASOUND TRAINING**

1. How did other providers receive the information from you when you had to communicate ultrasound results and/or a change in management plan? *Probe: how did doctors receive the information? Did you encounter any disbelief or doubt in your assessment?*
2. What do you remember the most about the training? *Probe: lecture? hands on practice? practice enrollments with a mentor? Consultant or TOT visits? Printed materials?*
3. What was the training strengths and weaknesses?
4. Which trainers did you feel most comfortable with and why? *Probe: ultrasound consultant(s); Master Trainer*
5. What other skill would you want to acquire in obstetric ultrasound?

Is there anything you would like to share that we didn’t get to talk about?

This is the end of the interview. Thank you very much for your time and for sharing your thoughts with us.

**Interviewer, note start time and stop audio-recording*

**Appendix W: HOSPITAL ADMINISTRATOR INTERVIEW GUIDE**

***Impact of introducing a clinical assessment checklist and limited obstetric ultrasound during labour triage to improve the detection of six high-risk obstetric conditions in select health facilities in Busoga Region, Uganda: a pre-post intervention study***

Hello, my name is *[state your name].* Thank you for your willingness to take part in this interview. I am here today to hear your thoughts about the introduction of ultrasound at your maternity ward. This interview should take approximately 30 minutes.

We are hoping you feel comfortable with us audio-recording this interview and taking some notes. None of your answers will be shared with your supervisors or colleagues at work. What you say will be shared with the ultrasound study team, but your name and location of work will not be connected to your answers in order to maintain your confidentiality. We appreciate your honest answers and respect all your opinions and thoughts. You can choose not to answer any of our questions at any time.

Date of interview: ______/ _______/ ____________

Venue: _______________________________

Designation of Respondent: ____________________

Interviewer _______________________

Time started: ________________

Time ended: _________________

Key informant interview identifier: __ *(KII/designation/date/cumulative no of KII done)* __________________________

**Interviewer, note start time and begin audio-recording*

1. What have you heard about the ultrasound study? *Probe: familiarity with study objectives*
2. Has the ultrasound study impacted your facility in any way? *Probes: provider desires to be part of the study; more women seeking care; opportunity to use the ultrasound in other departments*
3. What do you perceive to be the advantages of offering ultrasound at this health care level?
4. What are some of the challenges? *Probe: how has staff rotation, turnover, and electricity affected the facility’s ability to roll ultrasound out?*
5. What would you recommend for optimal implementation of ultrasound at this level? *Probe: dedicated personnel in the maternity ward to ultrasound (e.g. radiographer) rather than midwife; generator for stable electricity;*

This is the end of the interview. Thank you very much for your time and for sharing your thoughts with us.

**Interviewer, note start time and stop audio-recording*

**Appendix X: FIELD NOTES | STRUCTURED OBSERVATION**

The observer will document what happens after a woman presents with labor pains, specifically focusing on the chronological order of assessments completed, the time it takes to complete such activities, and general system factors that influence these workflow processes. Observations will stop at 90 minutes following presentation, or if the woman begins pushing within that time frame.

| Observer initials | |  | | | **OBSERVATION NOTES**  **ULTRASOUND TRIAGE STUDY** |
| --- | --- | --- | --- | --- | --- |
| Date | |  | | |
| Facility | |  | | |
| Patient arrival time/ observation start time | |  | | |
| Stage of labor at time of presentation | |  | | |
| **Observation time points** | | **Activities that occurred (please use activity codes below and include all that apply)** | | | **Additional contextual information about this time frame, including patient and provider demeanors, interruptions, etc.** |
| First 15 minutes of patient arrival | |  | | |  |
| between 16-30 minutes | |  | | |  |
| between 31-45 minutes | |  | | |  |
| between 46-60 minutes | |  | | |  |
| between 61-75 minutes | |  | | |  |
| between 76-90 minutes | |  | | |  |
| **ACTIVITY CODES** | | | | | |
| **1** | Timing of first contact with provider | | **13** | Lab diagnostics initiated | |
| **2** | Patient history taken | | **14** | Woman receiving medications (please specify, e.g. ACS, MgSO4, oxytocin) | |
| **3** | Pelvic exam conducted | | **15** | Woman admitted | |
| **4** | Maternal vital signs, including blood pressure | | **16** | Woman sent home | |
| **5** | Fetal heart rate checked by Pinard horn | | **17** | Woman began pushing | |
| **6** | Fetal heart rate checked by ultrasound | | **18** | Woman sent to theater | |
| **7** | Ultrasound scanning – presentation | | **19** | Woman referral to another facility initiated | |
| **8** | Ultrasound scanning – placental location | | **20** | Patient waiting | |
| **9** | Ultrasound scanning – fluid volume | | **21** | Provider filling out of paper work | |
| **10** | Ultrasound scanning – multiple gestation | | **22** | Woman sent to OPD for ultrasound | |
| **11** | Ultrasound scanning – gestational age fetal biometry measurements | | **23** | Mother ran away | |
| **12** | Provider consulting with other provider/specialist | | **24** | Other, please describe | |

Please comment on the prompts below in order to provide additional context during this observation period.

|  | **PROMPTS** | **NOTES** |
| --- | --- | --- |
| **Provision of ultrasound scan** | - Did a scan take place? - How long did the scan/checklist take? - Was it uninterrupted? |  |
| **Patient and provider interactions during the scan** | - Number of providers who consulted with the woman - Describe the communication between the provider(s) and patient. - Describe the patient’s demeanor. |  |
| **Estimated times** | - How many minutes did the provider spend with the woman? - How many minutes did the woman wait for care, results, etc? |  |
| **System factors** | - Overall patient load during this time - Number of midwives on staff - Concurrent services offered at the facility (e.g. ANC, PNC) - Any other issues affected workflow or context (e.g. power, weather) |  |
| **Any additional information?** |  | |
| **Observation end time** |  | |
